# Modeling of Hypo/Hyperglycemia and Their Impact on Breast Cancer Progression Related Molecules

**DOI:** 10.1371/journal.pone.0113103

**Published:** 2014-11-17

**Authors:** Sirin A. I. Adham, Hasina Al Rawahi, Sumaya Habib, Mansour S. Al Moundhri, Alicia Viloria-Petit, Brenda L. Coomber

**Affiliations:** 1 Department of Biology, College of Science, Sultan Qaboos University, P. O. Box 36, 123 Muscat, Oman; 2 Department of Medicine, College of Medicine, Sultan Qaboos University, P.O. Box 35, 123 Muscat, Oman; 3 Department of Biomedical Sciences, Ontario Veterinary College, University of Guelph, Guelph, ON, Canada; Wayne State University School of Medicine, United States of America

## Abstract

Breast cancer (BC) arises commonly in women with metabolic dysfunction. The underlying mechanism by which glycemic load can exert its action on tumor metastasis is under investigated. In this study we showed that glycemic microenvironment alters the expression of three classes of proteins, VEGF and its receptors, cell to cell, and cell to extracellular matrix (ECM) adhesion proteins in MDA-MB-231 parental cells and its two metastatic variants to the bone and brain (MDA-MB-231BO and MDA-MB-231BR, respectively). Using western blotting, we showed that VEGFR2 levels were higher in these variant cells and persisted in the cells under extreme hypoglycemia. Hypoglycemia did not alter VEGFR2 expression *per se* but rather suppressed its posttranslational glycosylation. This was reversed rapidly upon the restoration of glucose, and cyclohexamide (CHX) treatment demonstrated that this deglycosylated VEGFR2 was not a product of de-novo protein synthesis. VEGFR2 co-receptor Neuropilin-1 was up-regulated four-fold in all MDA-MB-231 cells (parental and two variants) compared to VEGFR2 expression, and was also susceptible to glycemic changes but resistant to CHX treatment for up to 72 hrs. Hypoglycemia also resulted in a significant decrease in specific catenin, cadherin, and integrin proteins, as well as cellular proliferation and colony forming ability. However, MDA-MB-231BR cells showed a unique sensitivity to hypo/hyperglycemia in terms of morphological changes, colony formation ability, integrin β3 expression and secreted VEGF levels. In conclusion, this study can be translated clinically to provide insight into breast cancer cell responses to glycemic levels relevant for our understanding of the interaction between diabetes and cancer.

## Introduction

Worldwide, Breast Cancer (BC) is considered the second most diagnosed type of cancer after lung cancer [1]. Metabolic disruption is an example of a recently described ‘emergent hallmark’ of cancer which indicates that cancer cells reprogram their metabolism in order to most effectively support their neoplastic proliferation [2]. Diabetes Mellitus (DM) and BC share many risk factors such as obesity, sedentary lifestyle, advanced age, and dietary risk factors (high intake of fat and refined carbohydrates) [3]. The two conditions that arise as a result of treating type II diabetes are hyperglycemia and hypoglycemia, which refer to chronically high and low blood glucose levels, respectively [4]. Metformin is a biguanide derivative which lowers the glucose levels in blood, having a protective effect against BC [5]. An epidemiological study showed that metformin also decreased the risk of BC by 19–66% when compared to non-treated diabetic cases [6]. Further specific studies defining the types and subtypes of BC on the molecular level will give insight into those BC patients who are responding differently to metformin treatment.

There are several hypotheses explaining the mode of how diabetes mellitus (with the coexistence of its complications, hyperglycemia and hypoglycemia), could exert effects on BC. It has been shown that the insulin-like growth factor IGF1 pathway is active in both BC and DM [7]. IGF1 is a mitogenic and anti-apoptotic agent, which activates pro-survival and proliferative pathways in normal breast cells, an action similar to estrogens in BC [8]. In addition to the activation of IGF1, insulin itself has mitogenic and anti-apoptotic effects on breast tissue through its activation of phosphatidylinositol 3-kinase (PI3-K), an important pathway in BC [9]. Recent reports stated the role of vascular endothelial growth factor (VEGF) in regulating cell metabolism. High plasma VEGF concentrations are associated with less carbohydrate intake and lower body mass in type II diabetes, and over expression of VEGF by the adipose tissue protects against diet-induced obesity and insulin resistance. In a recent report, VEGF neutralization resulted in improving the diet induced metabolic dysfunction in a mouse model [10], [11], [12]. IGF-IR was co-localized along with VEGF receptor 2 (VEGFR2) on circulating epithelial cancer cells of BC patients [13]. In general, breast cancer resistance to hormonal therapy has been linked with high activity/expression of receptor tyrosine kinases. In particular, the VEGF/VEGFR2 pathway supports the growth of estrogen-independent breast cancer cells [14].

Based on these previous observations we hypothesized that VEGFR2 expression in BC cells might be modulated by the changes in the glycemic tumor microenvironment and this modulation would depend on the site of metastasis. Previously we described how glucose concentration acts as a key regulator for VEGF receptor VEGFR2 in epithelial ovarian cancer (EOC) cells, where this protein was degraded by the proteosome under hypoglycemic conditions [15]. In this present study we investigated the effect of hypoglycemia and hyperglycemia on three major classes of proteins: (i) the ligand VEGF and its receptors (VEGFR1, VEGFR2 and co-receptor Neuropilin-1), (ii) cell-cell adhesion proteins, (iii) cell to ECM adhesion proteins. We evaluated these molecules in the MDA-MB-231 cells (MDA-MB-231P), and their metastatic variants to the bone (MDA-MB-231BO) and brain (MDA-MB-231BR) [16]. Unlike our previous findings with EOC cells, we found that VEGFR2 protein is not degraded but rather persists in the BC cells under hypoglycemia with a reduction in its posttranslational glycosylation. Glucose restoration led to its rapid re-glycosylation. The MDA-MB-231BR cells were the most affected by the glycemic changes, showing the most notable changes in protein expression in response to hypo/hyperglycemia and exclusive up-regulation of β3 integrin with higher concentrations of glucose.

Finally, our results show that the VEGF/VEGFR2 axis is differentially modulated by glycemic load among the cells studied. We suggest VEGFR2 modulation by glucose is important for the progression of BC cells. Breast cancer progression, particularly the hormone resistant types represented by MDA-MB-231 cells, might be linked with diabetes mellitus complications (hyper/hypoglycemia) through the VEGF pathway since under extreme hypoglycemic conditions VEGFR2 persisted in the cells, did not degrade and was rapidly switched to its mature functional form upon availability of glucose.

## Materials and Methods

### Cell lines and Cell Culture

MCF-7, BT-474, and Parental MDA-MB-231 cells were purchased from ATCC USA; bone (MDA-MB-231BO) and brain (MDA-MB-231BR) metastasized BC cell variants of MDA-MB-231 were previously derived and characterized [16] and were kindly provided by Dr. T. Yoneda (M.D. Anderson Cancer Center, Houston, Texas). The cells were grown in complete culture medium DMEM containing 10% fetal bovine serum (FBS, Sigma), 1% sodium pyruvate (Sigma-Aldrich) and 0.5% gentamycin (Invitrogen). They were grown and maintained in 5% CO_2_ in a humidified atmosphere at 37°C.

### *In* *vitro* Hypoglycemia, Hyperglycemia and Normoxia

When the cell lines reached 100% confluency in 10 cm plate, they were washed twice with PBS and the normal growth media was replaced with serum free media as follows: 10 ml special DMEM without glucose (0 mM; hypoglycemia) (Gibco, life technologies, Germany). The hyperglycemic condition was achieved by incubating the cells in 10 ml of usual DMEM-high glucose containing 25 mM of glucose and the normal physiological glucose concentration (5 mM) of DMEM was prepared as a combination of 2 ml from the standard DMEM high glucose (Sigma, Germany) which contains 4500 mg/ml of glucose (25 mM) and 8 ml of the 0 mM DMEM.

### Glucose Restoration after 24 h of Hypoglycemia

To study the expression pattern of VEGFR2 after the restoration of glucose the cells were initially grown in DMEM with 0 mM glucose for 24 h. After 24 hours the cells were re-exposed to regular DMEM (25 mM glucose) and samples were collected for western blotting after various times of glucose restoration. The total protein was extracted from the cells in the restored glucose conditions and their respective controls (cells not exposed to hypoglycemia for 24 h) at different time points, including 10, 20, 30, 40 and 50 minutes and 1, 2, 3, 4, 5, 6, 7, 8, and 12 h.

### Cyclohexamide Treatment after 24 h of Hypoglycemia and Restoration of Glucose

To check the effects of halting de novo protein synthesis in the cells, the cells were treated with 10 µg/ml of cyclohexamide (CHX; Sigma Aldrich) in two conditions: DMEM with 0 mM glucose (–glu) and DMEM with 25 mM glucose (+glu), and samples collected for western blotting. The respective controls were: +glu–CHX; –glu–CHX; +glu+CHX; –glu+CHX. These conditions were repeated in five different time points: 10 min, 20 min, and 1, 3 and 6 h upon glucose restoration.

### SDS-PAGE and Western Blotting

Total protein lysates were loaded equally in the wells of SDS-PAGE, and after electrophoresis, transferred to PVDF membrane (Millipore). Thereafter, the membranes were washed with TBS solution and blocked for 30 min in 5% free fat milk dissolved in 1X TBST (Tris-base, NaCl and 0.001% Tween-20 with pH 7.6). After blocking, the membranes were incubated overnight at 4°C with the corresponding primary antibody (Table S1) diluted in the same blocking solution. POD-conjugated secondary antibody and BM chemiluminescence western blotting substrate (Roche) were used to visualize bands on an X-ray sensitive film (Roche). Western blots were repeated at least three times and a representative blot from consistent triplicates was chosen for the figures. The graphs were prepared to represent the intensity of the bands on the x-Ray films which were quantified using the Image J software and the average of three independent replicates was calculated.

### Real -Time qPCR

RNA isolation from the different cell lines under the three different glycemic conditions (0 mM, 5 mM and 25 mM) was done using the Trizol reagent (Sigma-Aldrich, USA). One microgram of RNA was treated with DNaseI (Invitrogen, USA) and converted to cDNA using high-capacity cDNA Reverse Transcription kit (Life technologies, USA). The transcripts were quantified using Real-time qPCR performed on Applied Biosystems 7500 fast Real-Time PCR detection System (Applied Biosystems, life technologies, USA). The reaction mix contained 20 ng of the cDNA template, 2 µl of each primer (10 µM forward and reverse primers) and 10 µl Fast SYBR Green master mix 2X (Life technologies, USA). The primer sequences [17] to amplify E-cadherin were: Sense- 5′-GGAAGTCAGTTCAGACTCCAGCC-3′ Anti Sense- 5′ AGGCCTTTTGACTGTAATCACACC-3′ and the beta actin primers were also designed based on previously published primers [18]: sense 5′-GCTGTGCTACGTCGCCCTG-3 and anti sense’ 5′-GGAGGAGCTGGAAGCAGCC-3′. The PCR profile was as follows: 20 seconds at 95°C, followed by 40 cycles of 3 seconds at 95°C and 30 seconds at 60°C. The obtained Ct value of each gene of interest was normalized to the Ct of the reference genes as follows: Delta Ct = Ct_gene test_–Ct_endogenous control_; Delta Delta Ct = ΔCt _sample1_– ΔCt _calibrator_; RQ = Relative quantification = 2^−ΔΔCt^ this RQ value represents the fold of increase compared to the reference gene.

### Colony Formation Assay

The effect of hypoglycemia on the ability of the cells to form colonies was investigated by growing the MDA-MB-231P, MDA-MB-231BO and MDA-MB-231BR for 24 hours in hypoglycemia (1000 cells in each well of the six well plates). The cells were then washed and re-incubated in regular DMEM for two weeks, then visualized by staining with crystal violet solution (25% methanol and 0.5% crystal violet). The colonies were manually counted and quadruplicates of each dose were performed.

### Alamarblue Assay

Cell viability was determined using Alamarblue Assay (Invitrogen, USA), following the manufacturers protocol. Briefly, 25,000 cells were seeded in 96-well dishes in normal growth medium. Twenty-four hours later, cells were washed twice with DMEM, then 100 µl serum-free DMEM with (25 mM) or without (0 mM) glucose were added to each well. After 12 hr, Alamarblue reagent was added to each well in a final concentration of 10%. 4 hours later, the absorbance at 570 and 630 nm was determined, and Alamarblue reduction was calculated based on manufacturer’s instructions. Each experiment was repeated at least twice in quadruplicates.

### VEGF ELISA

To evaluate the quantity of VEGF secreted by the cells under the two conditions (hypo and hyperglycemia) the cells were seeded in 24 well plates in regular growth media. After 24 h the media was changed to DMEM with 1% FBS with either 0, 5, or 25 mM glucose and cells were grown for another 48 hr. Conditioned media samples were collected and subjected to quantification by ELISA for human-specific VEGF-A following the manufacturer’s protocol (R&D Systems, Minneapolis, MN, USA).

### Statistical analysis

Statistical analyses were carried out with GraphPad InStat 3 Software for parametric data and statistically significant differences were determined by two-tailed unpaired student’s t test or one-way ANOVA. Kruskal-Wallis H test was used for the analysis of the non-parametric data. Significant differences were defined as p<0.05.

## Results

### Different Levels of VEGFR2 in MDA-MB-231P and its Two Variants MDA-MB-231BO MDA-MB-231BR

**Figure 1:**
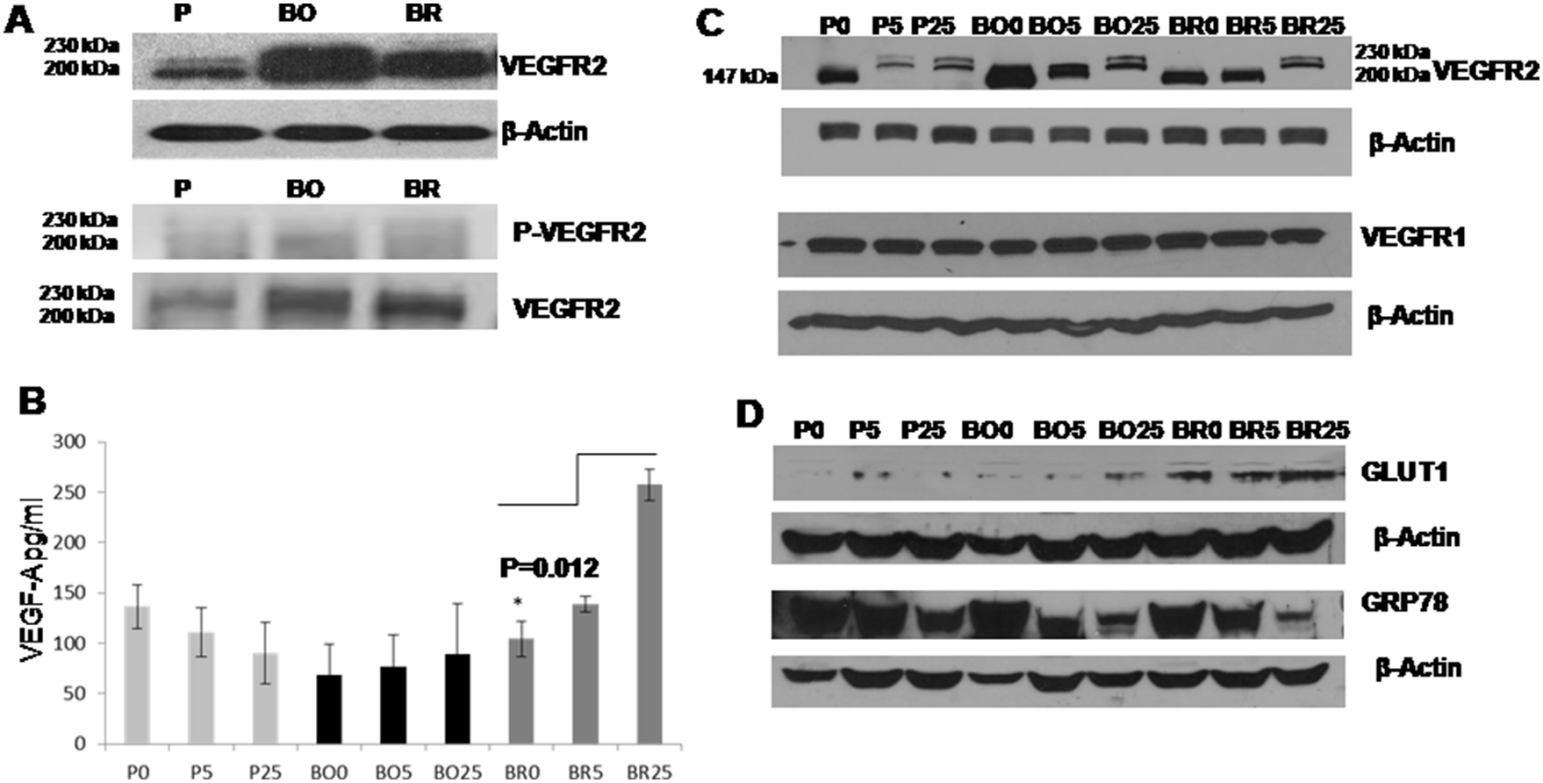
The expression of VEGF and its receptor VEGFR2 in MDA-MB-231 cells and its metastatic variants varies with glycemic conditions. (A) Western blots show the difference in VEGFR2 and phospho-VEGFR2 in MDA-MB-231 parental (P), bone metastatic (BO) and brain metastatic (BR) variants growing in DMEM containing 25 mM glucose. (B) Graph represents the mean of four independent ELISA experiments measuring the total VEGF-A secreted in the conditioned media, (error bars represent ± standard deviation). Statistical analysis showed no significant difference in the amount of VEGF-A secreted when the MDA-MB-231P and MDA-MB-231BO cells were grown under the different glucose concentrations. However, MDA-MB-231BR cells produced significantly higher levels of VEGF-A (p = 0.012) when grown in DMEM containing 25 mM of glucose compared to 0 mM of glucose. Figure abbreviations P: MDA-MB-231 Parental, BO: MDA-MB-231Bone and BR: MDA-MB-231Brain; cell lines were grown in DMEM 0, 5 and 25 mM glucose for 24 hrs without FBS. (C) VEGFR2 expression in MDA-MB-231P, MDA-MB-231BO and MDA-MB-231BR cells. The 147 kDa band was the dominant band expressed at 0 mM glucose. The regular double bands (200 kDa and 230 kDa) were detected in all the cells at 25 mM glucose. The cells expressed the bands differentially when placed in 5 mM glucose media. The MDA-MB-231P cells produced the 200 and 230 kDa forms; the MDA-MB-231BO cells produced the 147 and 200 kDa forms while the MDA-MB-231BR cells did not express any of the mature glycosylated bands at 5 mM glucose concentration. The bottom blot shows the expression of VEGFR1 was constant and did not change among the cell lines nor under the different glucose conditions. (D) Blot shows MDA-MB-231BR cells produced higher levels of GLUT-1 protein than the other cell lines. GRP78 had the same expression pattern among the different cells and its expression had an inverse relationship with glucose concentration. Note: β-Actin was used in all the blots as protein loading control.

Growing in DMEM (glucose levels 25 mM-FBS; i.e., hyperglycemia) breast cancer MDA-MB-231P cells expressed the least VEGFR2 when compared to their metastatic derivatives to the bone (MDA-MB-231BO) and brain (MDA-MB-231BR). The highest VEGFR2 expression was detected in the MDA-MB-231BO cell line and MDA-MB-231BR cells showed intermediate expression (**Figure 1A**). The phosphorylated VEGFR2 was detected in all the MDA-MB-231cells (parental and metastatic variants) only when the cells were growing in 25 mM of glucose (**Figure 1A**).

### Brain Metastasized Cells (MDA-MB-231BR) Showed a Significant Decrease in VEGF Secretion under Hypoglycemia

In four independent experiments, VEGF (pg/ml) was quantified and showed a non-significant difference in the levels secreted by MDA-MB-231P and MDA-MB-231BO cells under the three growth conditions (0 mM, 5 mM, 25 mM glucose) (**Figure 1B**). In hyperglycemic condition (25 mM of glucose) MDA-MB-231BR secreted significantly higher levels of VEGF (p = 0.0122) when compared with its VEGF secretion in hypoglycemia and when compared with the other cells under the same hyperglycemic conditions.

### VEGFR2 was Expressed in its Non-glycosylated Isoform after 24 Hours of Glucose Deprivation

The mode of VEGFR2 isoform expression was different among the three cell lines (parental and the two variants). Both the mature glycosylated form of the protein (230 kDa) and the immature unglycosylated form (200 kDa) were detected when all the cell lines were grown under hyperglycemic conditions (25 mM of glucose), (**Figure 1C**). In normal physiological glucose (5 mM) only the parental cells was able to produce the mature 230 kDa form of VEGFR2. The two metastasis derivative cell lines (MDA-MB-231BO, MDA-MB-231BR) produced only the immature glycosylated and unglycosylated forms (200 and 147 kDa, respectively) in DMEM with 5 mM glucose. In hypoglycemic conditions (0 mM) all the cells produced only a 147 kDa VEGFR2 as the unglycosylated form of the protein. All the MDA-MB-231 cells expressed VEGFR1 equally without any significant reduction in amount or molecular weight under hypo or hyperglycemia (**Figure 1C**). In addition to the higher levels of VEGF, MDA-MB-231BR cells also expressed higher levels of GLUT-1 protein when compared with the MDA-MB-231P and MDA-MB-231BO cells and in all the cells tested GLUT-1 protein increased when glucose concentration increased (**Figure 1D**). Finally, we also examined the involvement of the unfolded protein response back up mechanism under hypoglycemia. All cells showed no significant difference in the levels of the unfolded protein response represented by the level of GRP 78 protein which was proportionally increased when glucose concentration decreased (**Figure 1D**).

### Mature Glycosylation of VEGFR2 is Restored upon Glucose Re-exposure

**Figure 2:**
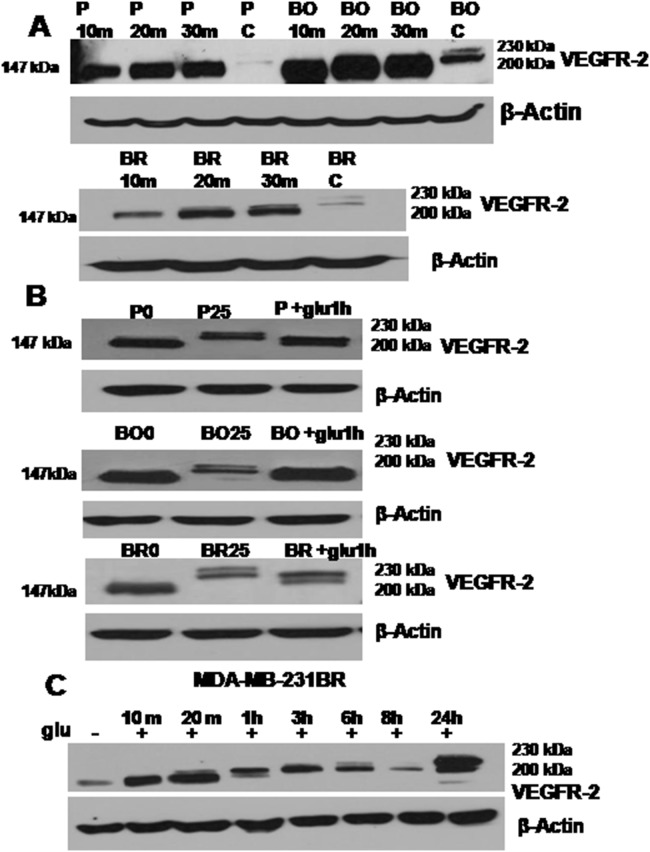
Glucose restoration resulted in a rapid glycosylation of the immature VEGFR2 band. (A) Western blot shows the VEGFR2 expression pattern in the cells exposed to 0 mM glucose media for a period of 24 hrs, followed by glucose restoration for 10, 20, 30 mins and the control cells (cells grown in control regular DMEM (25 mM glucose) without prior hypoglycemic exposure) P: MDA-MB-231P; BO: MDA-MB-231BO and BR: MDA-MB-231BR. The mature VEGFR2 band at 200 kDa was detected after 20 mins of glucose restoration which was more obvious in the MDA-MB-231BR (bottom blot). (B) Western blots showing that glucose restoration for 1 hour (after a period of 24 hrs of hypoglycemia) enhanced the formation of the glycosylated band of VEGFR2 (200 kDa) in all three cell lines MDA-MB-231P (P+glu 1hr), MDA-MB-231BO (BO+glu1h) and MDA-MB-231BR (BR+glu1h). The samples indicated by P25, B25 and BR25 are the samples collected from the cells growing in regular DMEM (25 mM glucose) expressing both mature bands of VEGFR2 (200 kDa and 230 kDa). P0, BO0 and BR0 are the MDA-MB-231P, MDA-MB-231BO and MDA-MB-231BR cells respectively, exposed to hypoglycemia for 24 hrs without glucose restoration. All three cell lines expressed only the unglycosylated band of VEGFR2 (147 kDa) when grown in 0 mM DMEM. (C) Western blots depicting the gradual increase in VEGFR2 band size upon glucose restoration in MDA-MB-231BR cells. Note: β-Actin was used in all the blots as a protein loading control.

Figure 2A shows that within 20 min of glucose re-exposure after 24 h hypoglycemia, the immature glycosylated 200 kDa isoform was detectable in all 3 cell types with the coexistence of the 147 kDa isoform. These two isoforms sustained their levels of expression until 1hr of glucose re-exposure (**Figure 2B**), after which the non-glycosylated (147 kDa) band was gradually lost (**Figure 2C**). With longer glucose re-exposure (approximately 4–6 hrs), the full mature VEGFR2 (230 kDa) and the immature 200 kDa bands were consistently detected (**Figure 2C**).

### The Recovery of Glycosylated VEGFR-2 upon Glucose Restoration was not a Product of de-novo Protein Synthesis

**Figure 3:**
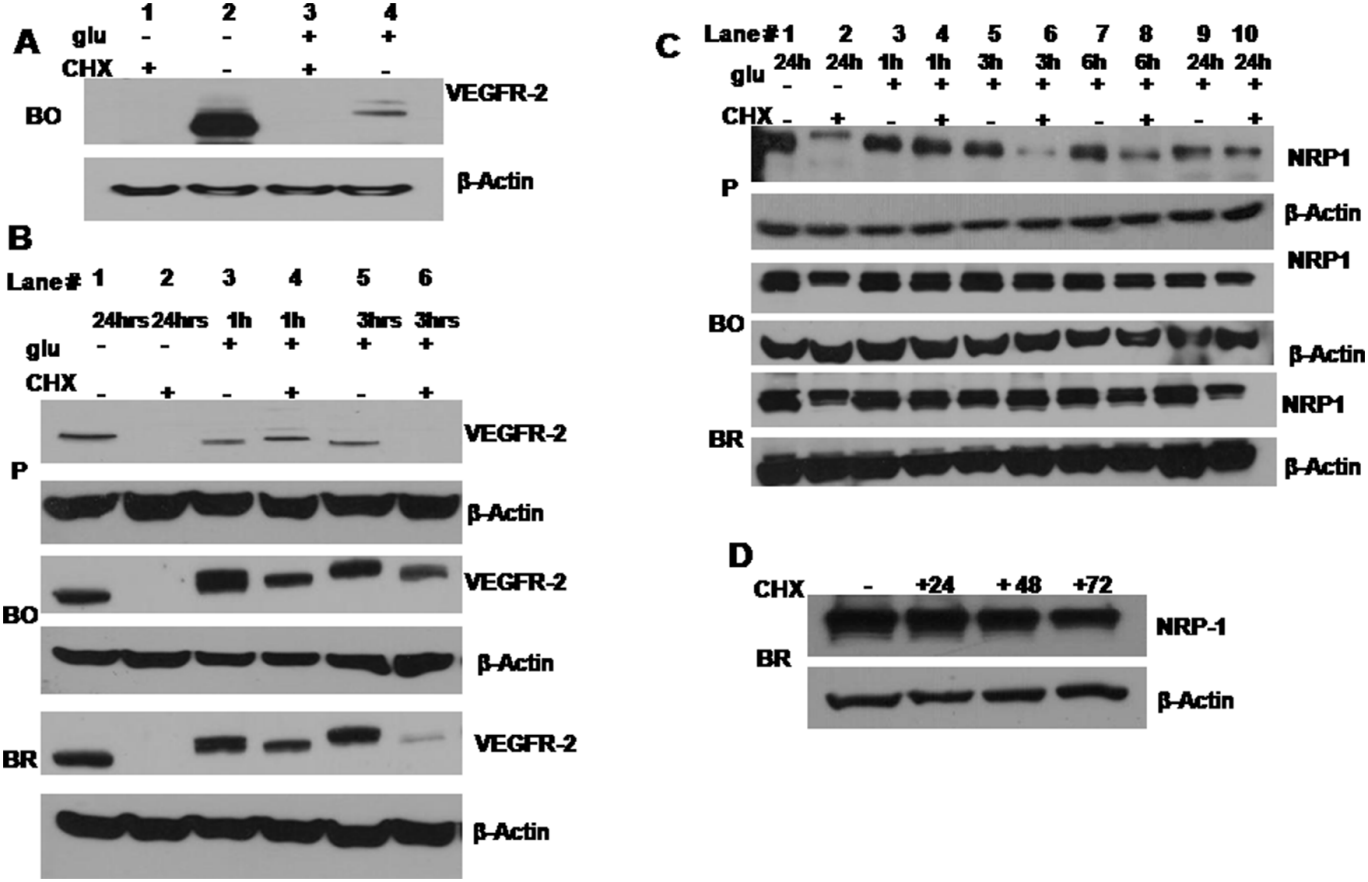
The glycosylated VEGFR2 found upon glucose re-exposure was not a product of a de-novo protein synthesis. (A) Cyclohexamide treatment for a period of 24 hrs depleted VEGFR2 protein from all three cell lines. The blot in this panel demonstrates that cyclohexamide depleted VEGFR2 from the MDA-MB-231BO cells (regularly expressing the highest level of VEGFR2 among the three cell lines) whether they were hypoglycemic or hyperglycemic (lane 1 and 3). The control cyclohexamide negative samples –glu −CHX and +glu -CHX showed detectable VEGFR2 expression (lane 2 and 4). (B) VEGFR2 was completely depleted from the three cell lines when grown in hypoglycemia in the presence of CHX (lane 2). The samples in lanes 3 and 5 are cells exposed to hypoglycemia for 24 hrs without CHX and glucose was restored for 1 hr (lane 3) or 3h (lane 5), respectively. Comparing the former two lanes (3 and 5) shows clearly that the VEGFR2 band was increasing in molecular weight as the time of glucose exposure increased; this is very prominent in the middle (MDA-MB-231BO) and bottom blots (MDA-MB-231BR). The samples in lane 4 and 6 were glucose restored for 1 hr and 3 hrs respectively with CHX added at the time of glucose restoration. After 3 hr of CHX and glucose restoration of MDA-MB-231P cells, the VEGFR2 band was not detectable (lane 6- P), however the MDA-MB-231BO and MDA-MB-231BR cells (lane 6 BO, lane 6 BR) expressed a detectable VEGFR2 band. Note: β-Actin was used in all the blots as a protein loading control. (C) Stability of NRP-1 expression under low glucose and CHX treatment. NRP-1 was detected in the same samples as in the previous panel (B) and it did not show a profound decrease in its expression in both MDA-MB-231BO and MDA-MB-231BR. However in MDA-MB-231P samples the NRP-1 band pattern was changed after 24 hrs of hypoglycemia in the presence of CHX (lane 2). The NRP-1 band was also decreased in the glucose-restored samples with CHX (added at the time of restoration) (lanes 6, 8, and 10). (D) The blot shows that in MDA-MB-231BR cells the NRP-1 protein was resistant to CHX treatment for up to 72 hrs of exposure. Note: β-Actin was used in all the blots as a protein loading control.

We used cycloheximide (CHX) treatment to confirm that VEGFR2 was not degraded during the hypoglycemic period and then re-synthesized upon glucose restoration. Initially, the effectiveness of cyclohexamide to block VEGFR2 synthesis was evaluated by growing the MDA-MB-231BO cells for 24 hrs in hypoglycemia (0 mM) and hyperglycemia (25 mM) in the presence or absence of CHX. CHX resulted in the depletion of VEGFR2 completely from the cells when exposed to 0 mM and 25 mM glucose. In contrast, in control conditions (without CHX) cells expressed the mature and immature glycosylated forms of the protein (230 kDa and 200 kDa, respectively) at 25 mM glucose and only predominantly the 147 kDa form at 0 mM of glucose (**Figure 3A**). VEGFR2 stability was detected after 1 hr and 3 hrs of glucose restoration with or without CHX. The VEGFR2 band intensity was reduced in the samples from CHX exposed cells and the band disappeared in MDA-MB-231P after 3 hrs of glucose restoration grown in the presence of CHX (**Figure 3B**). In control samples in which glucose was restored and CHX was not added, VEGFR2 band size was proportionally increasing with time indicating that the protein was not degraded and only its glycosylation status was altered. This was particularly clear in the two variant cells since they express higher levels of VEGFR2 (**Figure 3B**).

### Neuropilin-1 (NRP-1) Expression was Highly Expressed in MDA-MB-231P and its two Variants and was Resistant to CHX Treatment

NRP-1 glycoprotein is a co-receptor for VEGFR2. It was found to be highly expressed in all the MDA-MB-231 cells used as only 25 µg of total protein lysate were loaded in the gel to get a reasonable signal while 100 µg was used to visualize VEGFR2 in the same samples. Under 24 hrs of hypoglycemia and the presence of CHX all cells (parental and variants) showed a decrease in the unglycosylated form of the protein (100 KDa band), however the 140 KDa form persisted. (lane 2 in **Figure 3C**). MDA-MB-231P cells showed a slightly different response to CHX- when the glucose was restored gradually to the cells, CHX resulted in a decrease in NRP-1 band intensity after the third hour of glucose restoration in the presence of CHX (**Figure 3C**). There were no differences in the NRP-1 band intensity when the cells were in hypoglycemia for 24 hrs and when the glucose was restored for another 24 hrs in the presence of CHX in both variant cells (lane 2 and 10, **Figure 3C**). Prolonged treatment of MDA-MB-231BR cells with CHX demonstrated that NRP-1 expression was not susceptible to degradation even after 72 hr of CHX treatment (**Figure 3D**), and the MDA-MB-231BO cells showed the same pattern (data not shown).

### Hypoglycemia Results in Changes in MDA-MB-231BR Cell Morphology

**Figure 4:**
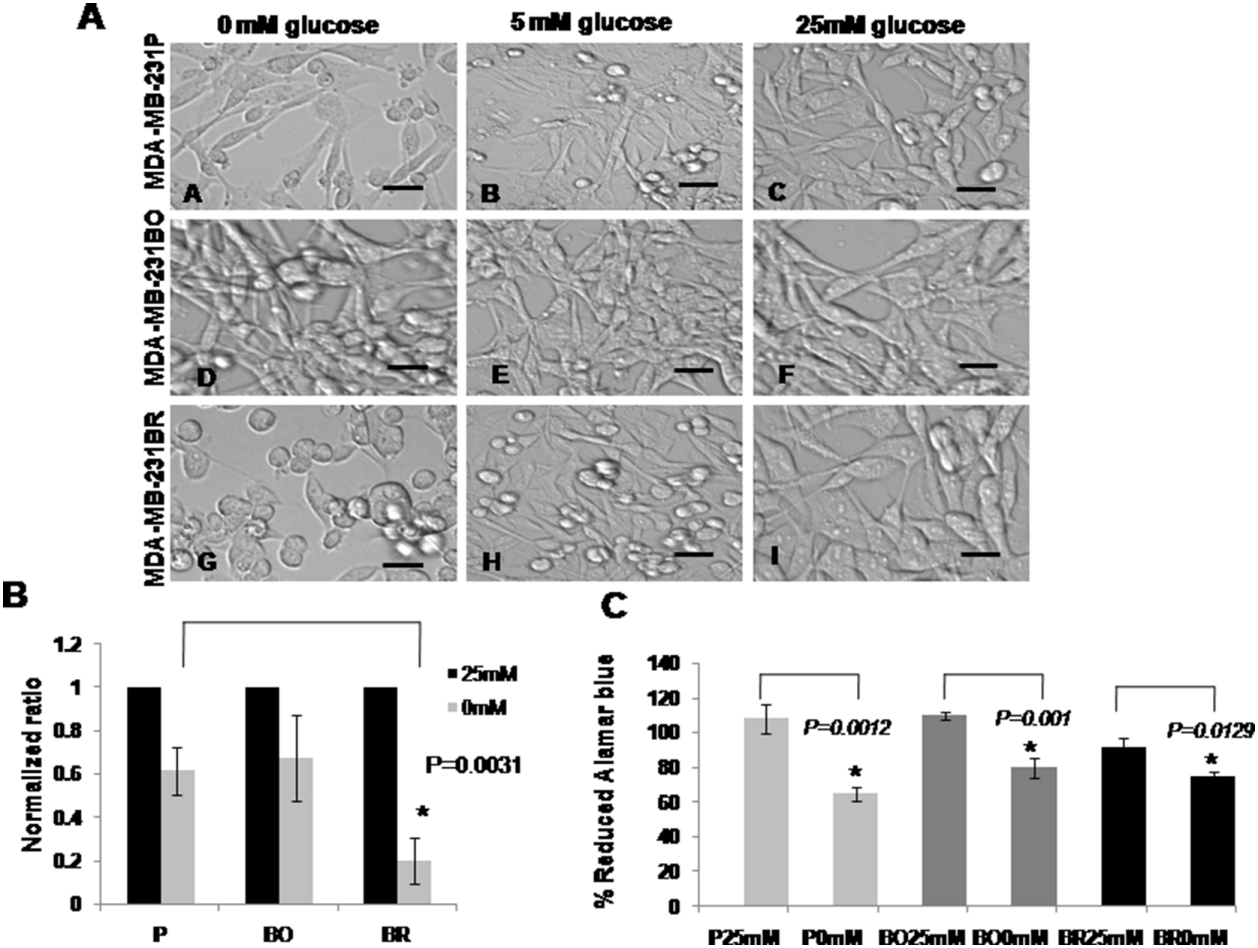
Hypoglycemia caused a change in MDA-MB-231BR cell morphology. Phase contrast images of the three MDA-MB-231 cell lines. Image (A) shows MDA-MB-231P cells grown in DMEM 0 mM glucose images; (B–C) the same cells grown in DMEM with different glucose concentrations: 5 mM and 25 mM. Image (D) represents MDA-MB-231BO cells growing in DMEM 0 mM glucose; images (E–F) are for the same cells grown in the other glucose concentrations. Neither MDA-MB-231P nor MDA-MB-231BO cells were morphologically changed by the various changes in glucose concentration. The MDA-MB-231BR cells grown in the absence of glucose for 24 hrs lost their extended protrusions (G), became rounded and maintained their attachment to the culture plate; images (H and I) show how the shape of the cells was restored upon the increase in glucose concentration. Scale bars = 100 µm. Breast cancer cells formed fewer colonies and had reduced proliferation under hypoglycemic conditions. (B) Graph represents the ratio of the average number of colonies counted in four independent experiments. The y-axis represents the normalized number of colonies counted in the hypoglycemia exposed wells versus the control formed by the cells. MDA-MB-231BR cells produced the least colonies among the three cell lines when exposed to hypoglycemia and this was confirmed by running the non-parametric Kruskal Wallis test H = 15.66 and p = 0.0013. (C) The graph represents cell growth as the percentage of Alamarblue reduced by the cells under hypo/hyperglycemia. There was a significant decrease in the reduced Alamarblue when the cells were grown in hypoglycemia for 24 hrs (MDA-MB-231P, MDA-MB-231BO, and MDA-MB-231BR) Results are shown as mean ± SD and were statistically analyzed by a two-tailed student’s t test (n = 3).

The morphology of MDA-MB-231BR variants changed upon glucose deprivation. Most cells lost their normal elongated shape and gained a rounded morphology, and as glucose concentration increased these cells gradually restored their normal shape (**Figure 4A**). The morphology of MDA-MB-231P and MDA-MB-231BO cells was not affected by glucose reduction (**Figure 4A**). The addition of FBS had no effect on cell morphology when cells were grown with 25 mM glucose (data not shown) demonstrating that glucose was the main factor behind this change.

### Hypoglycemia Reduced the Colony Formation Ability of Breast Cancer Cells

Next, we evaluated the effect of hypoglycemia (24 hrs 0 mM glucose) on the ability of MDA-MB-231 parent cells and variants to form colonies. The colonies formed were different among the three cell lines; MDA-MB-231BO and MDA-MB-231BR cells produced large and well-delimited colonies compared to the MDA-MB-231P cells which formed smaller, disorganized colonies. Glucose deprivation for 24 hrs and its restoration with regular DMEM (25 mM glucose) reduced the ability of all MDA-MB-231 cells to form colonies when compared with the cells not exposed to hypoglycemia (**Figure 4B**). The statistical analysis of four independent experiments showed that a significant decrease in colony number was observed mainly in the MDA-MB-231BR cell line p = 0.0013 (**Figure 4B**).

### Hypoglycemia Resulted in a Significant Decrease in the Cells Proliferation Ability

The proliferation ability of the MDA-MB-231 cells was determined using the Alamarblue assay. In general, all cells (MDA-MB-231P, MDA-MB-231BO, and MDA-MB-231BR) showed significant decrease in the percentage of reduced Alamarblue when exposed to hypoglycemia; p values were 0.0012, 0.001 and 0.0129 respectively (**Figure 4C**). The results represent the mean of three independent experiments.

### Profiling the Expression of Adhesion Molecules under Hypoglycemia

**Figure 5:**
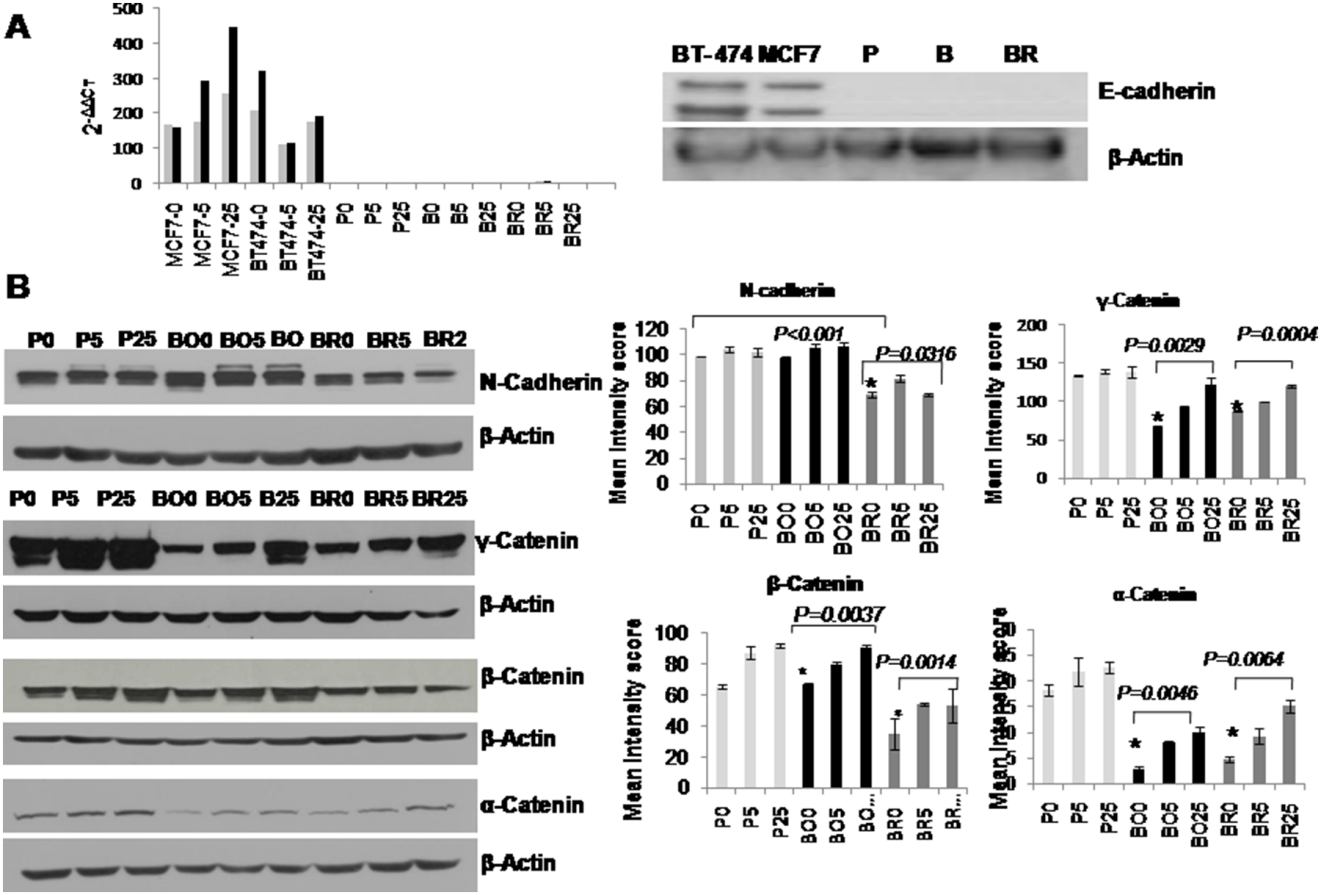
Expression of E-Cadherin, N-Cadherin, γ, β and α-Catenins in response to glucose for 24 **hrs.** (A) The graph (left) shows real time PCR results for the positive expression of E-cadherin in MCF-7 and BT-474 cell lines; the grey and black bars represent two repeated independent experiments. However, MDA-MB-231P and its two variant cell lines did not express detectable E-cadherin message or protein (right); (B) Representative blots (left) and quantification (right) shows that MDA-MB-231P and MDA-MB-231BO cells expressed higher levels of N-cadherin compared with the MDA-MB-231BR cells, and that N-cadherin in the former cells was significantly reduced under hypoglycemia. γ, β and α-Catenins were all reduced under hypoglycemic conditions and their expression was significantly decreased in the two MDA-MB-231 variant cells only. Error bars represent ± standard deviation. Note: β-Actin was used in all the blots as a protein loading control.

Given the morphological changes observed in the MDA-MB-231BR metastatic variant upon hypoglycemic conditions, we screened all cells for their expression of a panel of cell adhesion molecules, which were previously shown to modulate cellular attachment and cell morphology, and influence metastatic ability. These molecules included the epithelial molecules E-cadherin, α, β and γ catenin, and the mesenchymal marker N cadherin (**Figure 5**). Consistent with previous studies which found that loss of E-cadherin associates with metastatic ability [19], MDA-MB-231P, MDA-MB-231BO and MDA-MB-231BR cells had completely lost E-cadherin expression (**Figure 5A**). N-cadherin was highly expressed in all the studied cells (only 10 µg of total protein lysate was loaded), and was down regulated in the MDA-MB-231P and MDA-MB-231BO cells under hypoglycemia. The MDA-MB-231BR cells expressed the lowest level of N-cadherin when compared to the other cells (**Figure 5B**; blot on the left and graph on the right of the panel). Expression of the known tumor suppressor γ-catenin was less in the metastatic variant cells when compared to the MDA-MB-231P cells and was also down regulated in hypoglycemia. β-catenin was detected in slightly higher levels in both MDA-MB-231P and MDA-MB-231BO when compared to MDA-MB-231BR, and was not significantly down regulated under hypoglycemia in MDA-MB-231P cells however its expression in both MDA-MB-231BO and MDA-MB-231BR cells was significantly reduced under hypoglycemia (p = 0.0037 and p = 0.0014) (**Figure 5B**; bottom left graph). α-catenin was the least abundant among the three catenins and was detected in slightly higher amounts in the MDA-MB-231P cells, with no significant change in response to glucose concentrations, however, it showed significant reduction under hypoglycemia in both variant cells (**Figure 5B**; bottom graph to the right).

### Integrin β3 was Exclusively up Regulated in the Brain Metastatic Variant Cells

**Figure 6:**
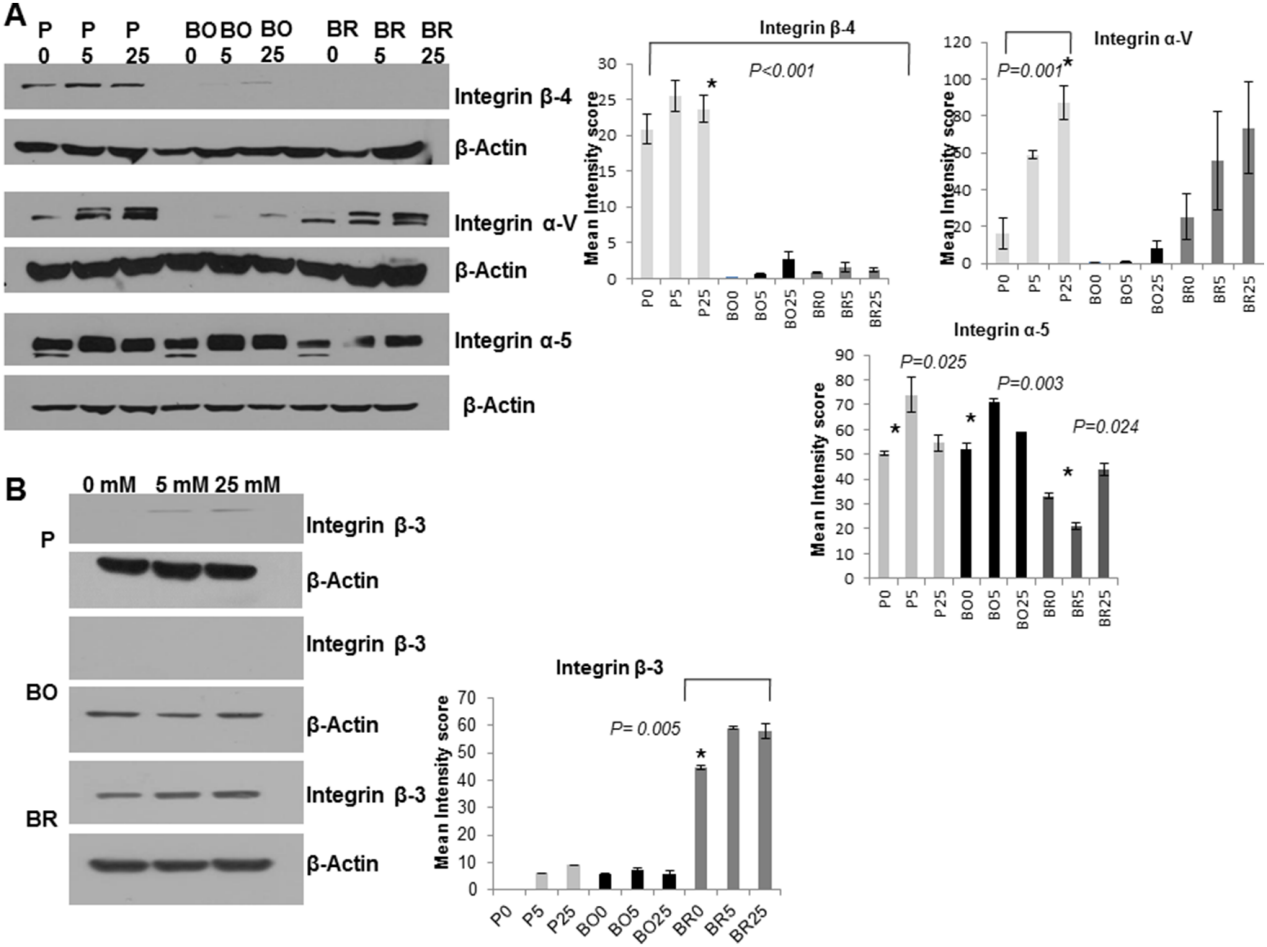
Integrin β4, αV, α5 and β3, were differentially regulated among the three cell lines in response to glucose. (A) Integrin β4 was weakly expressed, or undetectable in the MDA-MB-231BO and MDA-MB-231BR cells respectively however, it was expressed in all glycemic conditions in MDA-MB-231P cells which was significantly higher than the variants cells p>0.001 (graph on the right). Integrin αv was down regulated in MDA-MB-231BO and not detected when cells were exposed to DMEM 0 mM glucose (B0) (graph on far right). Integrin α-V was significantly decreased under hypoglycemia only in MDA-MB-231P cells p = 0.001. Integrin α5 was expressed in all the three cell lines and was down regulated in the absence of glucose in all the cells in study (bottom graph on the right and lower left panel). Integrin β3 was not detectable in the MDA-MB-231BO cells, and barely detectable in the MDA-MB-231P cells. It was expressed in detectable levels in MDA-MB-231BR cells and was slightly down regulated in reduced glucose. Error bars represent ± standard deviation. Note: β-Actin was used in all the blots as a protein loading control.

The expression of cell-to-ECM adhesion molecules, namely integrins, β4, αV, α5, and β3 were examined in our cell lines. β4 integrin was detected at higher levels in the MDA-MB-231P cells (with no obvious reduction under hypoglycemia), expressed at very low levels in MDA-MB-231BO cells, and almost not detected in MDA-MB-231BR cells (**Figure 6A**; blot on the left and graph on the right of the panel). Integrin αV expression was lower in MDA-MB-231BO cells as compared to MDA-MB-231P and MDA-MB-231BR cells, and was reduced in all studied cells in response to hypoglycemia however, this reduction was only significant in MDA-MB-231P cells. Integrin α5 was highly expressed in all the cells (**Figure 6A**), MDA-MB-231BR cells exhibited higher levels of integrin β3 as compared to the other two cell variants and the expression of this integrin was significantly reduced under hypoglycemia in MDA-MB-231BR cells p = 0.005 (**Figure 6B**; blots on the left and graph on the bottom right).

## Discussion

Hypoglycemia and hyperglycemia are important states that affect the tumor microenvironment and they usually arise as the manifestation of diabetes mellitus [20]. In this study we aimed to verify the response of MDA-MB-231P, MDA-MB-231BO, and MDA-MB-231BR cells to define special aspects of protein expression patterns in an attempt to determine targets that are affected by the surrounding microenvironment. The results of this study eventually will aid in understanding the mechanisms relating the complications of diabetes mellitus with BC progression and metastasis. In this present *in* *vitro* comparative study we found that the expression of VEGFR2 in MDA-MB-231P cells was less than that in the metastatic derivatives MDA-MB-231BR and MDA-MB-231BO, respectively. This suggests that VEGFR2 expression may be reflective of the invasion and progression stage of breast cancer [21]. This finding is consistent with our previous report in which we indicated that VEGFR2 expressed by cancer cells supports tumor cell proliferation and cell survival in suspension [22]. According to several reports MDA-MB-231 does not express the receptors for estrogen, progesterone nor human epidermal growth factor receptor 2 (ER, PR, HER2, respectively), and they were isolated from a hormone resistant tumor [23], [24] therefore, we investigated the impact of glycemic load on the expression of VEGF/VEGFR2 which is known to support the growth of this molecular subtype of breast cancer cells [14].

VEGFR2 is initially synthesized as a 147 kDa protein, and after posttranslational glycosylation the protein matures to become a 200 kDa molecule with further glycosylation bringing the protein to its full mature size which is 230 kDa; both the 200 and 230 kDa isoforms can be activated by phosphorylation [25]. We show here that hypoglycemia alone is not effective at halting the synthesis of VEGFR2, which was constantly expressed in its immature form under hypoglycemia. Restoration of glucose allows it to develop into its mature state through posttranslational glycosylation. We found the two metastatic variant cells had higher levels of VEGFR2 than the parental cells, and the levels were different between them. The MDA-MB-231BO had more VEGFR2 than the MDA-MB-231BR cells, indicating that the metastatic site may influence expression of VEGFR2 protein by breast cancer cells [26]. We know that this particular molecular subtype of breast cancer expresses high levels of VEGFR2 protein which is proportionally related with hormone resistance [27]. These results might be translated clinically to answer the question why the glucose reducing agent metformin works as a protective agent against breast cancer progression in some patients and not in all. Thus triple negative breast cancer patients who are difficult to treat with hormone therapy might be treated with a combination of anti-VEGF therapy and metformin, which collectively could reduce the burden of these tyrosine kinases.

Neuropilin-1 was highly expressed in all the cells and was more stable than VEGFR2 under hypoglycemia. We previously showed knockdown of VEGFR2 in EOC was accompanied by an increase in NRP-1 expression and was associated with a more aggressive phenotype [28]. We also showed hypoglycemia resulted in diminishing NRP-1 expression from EOC [15]. The sustained NRP-1 expression under prolonged periods of hypoglycemia in the breast cancer cells used here suggests that this protein may play an important albeit different role in these cells as compared to EOC cells. NRP-1 has a long half-life in the MDA-MB-231 cells; its levels only started to decrease slightly after 72 hrs of CHX exposure, while VEGFR2 was completely degraded after 3 hrs of CHX treatment. This indicates that the two proteins (VEGFR2, NRP-1) are differentially regulated by glucose in MDA-MB-231 cells. Brain metastatic MDA-MB-231 variant cells showed unique features in response to hypoglycemia such as a rounded morphology and an increase in GLUT-1 protein expression. These observations are in agreement with previous findings that alterations in cell morphology accompany changes in the metabolic activity of cancer cells [29]. The changes we observed in MDA-MB-231BR cells suggest that the metabolic and biochemical profile of breast cancer cells might influence which site they are able to colonize. Alternatively, microenvironmental features of different tissue beds may select for cancer cells with specific features to exploit such a niche. A recent finding showed that brain metastasized breast cancer cells express γ-aminobutyric acid (GABA)-related genes which enables breast cancer cells to utilize GABA as an oncometabolite, this represents neuronal like adaptation mechanism by which breast cancer cells transforms to brain [30].

Previous study indicate that hypoglycemia but not hyperglycemia increased glucose transport across blood-brain barrier endothelial cells present in the CNS; this was mainly due to increased GLUT1 on the luminal surface of the cells [31]. MDA-MB-231BR cells were unique in up-regulating the GLUT1 protein in a glucose dependent fashion compared with the other two cell lines, indicating their possible adaptation to the new location where surrounding brain tissue is subjected to high glucose [32]. However, GLUT1 protein expression was slightly down regulated upon hypoglycemia in MDA-MB-231BR cells which contradicts what has been found in endothelial cells [31]. In cancer cells, cell morphology reflects gene expression and patterns of protein expression which correlate with tumor cell invasiveness [33]. In monolayer cultures MDA-MB-231 cells have a bipolar spindle shape with extended protrusions at both ends of the cell body [34]. We found the morphology of MDA-MB-231BR cells was the most sensitive to glucose alteration in the culture media. When glucose was absent, the cells lost their normal morphology and became rounded without the extended protrusions and when glucose was restored the cells reverted to their usual morphology. Similar morphological reversibility was observed in MDA-MB-231 and MCF-7 cells when treated with inhibitors for β1 integrin, PI3K and MAPK which resulted in nearly complete phenotypic reversion in three dimensional contexts [35].

In MDA-MB-231BR cells we noticed that integrin β3 was exclusively up regulated compared to the other cell lines, and hypoglycemic conditions resulted in its down regulation. This finding can be explained by the previous report which showed that integrin αvβ3 activation in MDA-MB-435 human cancer cells (a highly metastatic cell line to brain) supports the efficient brain metastatic growth through continuous up-regulation of VEGF protein under normoxia [36]. We detected αv protein in both MDA-MB-231P as well as MDA-MB-231BR variant cells and it was regulated by glucose, while MDA-MB-231BO variant cells expressed barely detectable levels under both physiological and hyperglycemic conditions. This result might indicate that although αv and β3 integrins form a heterodimer, integrin β3 alone or in another complex might be enough to drive the metastasis to brain. Further analysis is required to evaluate this observation.

The cell-cell adhesion molecules tested were also differentially regulated in MDA-MB-231 and its variant cells. For instance there was a profound decrease in γ-catenin protein in the metastatic derived cells and its expression was increased in hyperglycemia, while β-catenin had a different response to glucose in the different cells. It has been reported that Wnt/β-catenin pathway and thioredoxin-interacting protein (TXNIP) mediate the “glucose sensor” mechanism in MDA-MB-231 cells, and higher glucose levels resulted in an increase in GSK3β and consequently higher levels of activated β-catenin protein [37]. Thus, our findings open a new avenue to determine the status of Wnt/β-catenin pathway in the MDA-MB-231 and its variants. Among the three cell lines studied, MDA-MB-231BR cells had the lowest capability to reduce Alamarblue and produced the fewest colonies after exposure to hypoglycemia. As a general observation, cancer cells use anaerobic glycolysis for their rapid growth, and thus are sensitive to glucose deprivation [38], [39]. It has been shown that the survival of MCF-7/ADR breast cancer cells was decreased exponentially up to 8 hours of their incubation in glucose-free medium due to the induction of c-myc dependent apoptosis [40]. However, in an in vivo breast cancer cell model for brain metastasis in immunodeficient mice, the MDA-MB-231BR cells were 2-fold less sensitive to the glucose analogue 2DG (modeling a glucose deprivation state) when compared to the MDA-MB-231P and MDA-MB-231BO variant cells [41].

We found that in hyperglycemia but not hypoglycemia, MDA-MB-231BR cells secreted higher levels of VEGF, which was different from the expression response of the MDA-MB-231P or MDA-MB-231BO. Another study found that up-regulation of vascular endothelial growth factor (VEGF) lead to activate αvβ3 integrin, which is important for the metastasis of breast cancer cells to the brain under normoxic conditions [36]. This may in part explain our results that MDA-MB-231BR cells produced higher levels of VEGF as well as higher levels of β3-integrin under the regular growth conditions. Our findings open new avenues for further biochemical and metabolic analysis to investigate the pathways that are activated or inactivated in response to glucose deprivation in brain metastasized breast cancer.

## Conclusions

The results of this study indicate that these two glycemic (hypo/hyperglycemia) conditions are controlling several pathways in these breast cancer cells differentially depending on their tissue localization preferences. Generally, hypoglycemia caused a negative effect on the proliferation and colony formation ability of MDA-MB-231 breast cancer cells. Hypo/hyperglycemia also had an impact on VEGFR2 and NRP-1 expression which supports the existence of a possible molecular correlation between type II diabetes mellitus and breast cancer progression. Hypoglycemia created a unique expression pattern of some adhesion molecules and tumor growth factors critical for their signaling pathways. Furthermore, the obvious morphological changes in MDA-MB-231BR cell line under hypoglycemia could be a result of lower levels of integrin β3. Finally, this translational research aids in shedding more light on the molecular targets for the different BC stages and tumor microenvironment which impact their progression.

## Supporting Information

Table S1 Primary antibodies used with their corresponding manufacturer and working dilution and the species raised in.
(DOC)
