# Supplementary material for: Modeling of Hypo/Hyperglycemia and Their Impact on Breast Cancer Progression Related Molecules
Source: PLoS One. 2014 Nov 17;9(11):e113103. doi: 10.1371/journal.pone.0113103 (PMC4234670; doi:10.1371/journal.pone.0113103)
Supplement: Table S1 — Primary antibodies used with their corresponding manufacturer and working dilution and the species raised in. (DOC) [file pone.0113103.s001.doc]

Table S1: Primary antibodies used with their corresponding manufacturer and working dilution and the species raised in.

| **Primary Antibodies** | **Species** | **Dilution** |
| --- | --- | --- |
| VEGFR2 (Cell Signaling) | rabbit | 1:1000 |
| E-Cadherin (Epitomics) | rabbit | 1:1000 |
| N-Cadherin (Epitomics) | rabbit | 1:2500 |
| Neuropillin-1 (Epitomics) | rabbit | 1:1000 |
| β-actin (Cell Signaling) | rabbit | 1:1000 |
| Phospho-VEGFR2 (Cell Signaling) | rabbit | 1:1000 |
| Integrin β3 (BD Biosciences) | mouse | 1:5000 |
| Integrin αV (BD Biosciences) | mouse | 1:500 |
| Integrin α5 (BD Biosciences) | mouse | 1:5000 |
| Integrin β4 (BD Biosciences) | mouse | 1:1000 |
| α-Catenin (BD Biosciences) | mouse | 1:500 |
| β-Catenin (BD Biosciences) | mouse | 1:1000 |
| GRP-78 (Santa Cruz Technology) | rabbit | 1:1000 |
| Glut-1 (Santa Cruz Technology) | rabbit | 1:1000 |
